# Should Anæsthesia Be Resorted to for the Extraction of Teeth

**Published:** 1883-06

**Authors:** J. H. Coyle

**Affiliations:** Thomasville, Ga.


ARTICLE III.

### SHOULD ANÆSTHESIA BE RESORTED TO FOR EXTRACTING TEETH?

BY J. H. COYLE, D. D. S., THOMASVILLE, GA.

"Knowledge hath indeed linked lands remote with bands of steel and made the lightning man's daily messenger; but her noblest triumphs lie not here. Even death has seen his might foreshortened, *Despair* and *Agony* are vanquished and Science hath *fettered pain*."—*Burns*.

If there is any one fact which has impressed itself on my mind over any other, in connection with dental practice of to-day, it is opposition to Anæsthesia by dentists; and I confess that I am at a loss to account for it. Surely it cannot arise from any lack of information as to the nature of Anæsthesia and its magic power for good, for they have open to them the same avenues to this knowledge as the medical profession, and, added thereto, a daily experience, should they adopt it in their practice. I am forced to the conclusion that, in a great majority of instances, it is the outcome of timidity, and this timidity arises from two sources: first, want of that experience which alone can ever give that *confidence* necessary; and, secondly, fear of adverse criticism.

Everywhere and in everything we read on the subject of Anæsthesia from a medical standpoint it is stated that

"these agents are not safe in the hands of dentists *who place their patients in a sitting posture.*" Even as late as last December, Dr. Chisholm uses that language in a paper read before the Baltimore Academy of Medicine. Surely all dentists do not administer Anæsthesia in a sitting posture. I know that *I do not, and never did.* Teeth can be as expeditiously and safely extracted in the recumbent position as any other surgical operation. However, assertions like this emanating from the medical press, coupled with an occasional report of a fatal chloroform norcosis, the agent administered *by a medical man at that,* with the patient in a dentist's chair, so operates on the fears of the mass of practicing dentists as to make them withhold from their patients this *priceless jewel, Anæsthesia.* They readily lend a listening ear to catch the repeated stories of its dangers, and close them to the testimony of its safety by those who have had *at least equal experience.*

Gross, Chisholm and many others who might be named, have testified that, in a life-time experience, covering thousands upon thousands of cases, that they have never had a fatal case in the use of what is considered the most powerful of these agents, even *chloroform.* In a conversation with Dr. Colton of New York City recently, he stated that in *one hundred and thirty-three thousand recorded cases of administration of nitrous oxide gas,* he had met with *not a single death,* or anything approaching it, only occasionally slight nausea produced. And so the list of favorable criticism might be extended, but it seems to me that it is already sufficient to carry conviction to a fair mind that Anæsthesia *intelligently produced,* is as safe as the exhibition of most substances enumerated in the Materia Medica.

For nearly twenty years I have never in a single instance refused Anæsthesia to any who have applied for it—making no discrimination whatever, as to age, sex or condition. I have produced Anæsthesia with chloroform in patients who had undoubted lesions of both heart and lungs, *without the semblance even,* of any outward symptom. I have always considered that *when I fonnd a fit subject for an operatton, I had also found a fit subject for Anæsthesia.* In the administration of chloroform I have always followed the following simple rules, which, if carefully observed, I am confident will bring the same good results to others:

First, if possible, have the stomach empty, by instructing the patient to abstain from the usual breakfast. Second, see that the clothing nor anything else does not bind the neck or chest, thus securing unobstructed circulation and a free play of the lungs. Third, give from one to two ounces of spirits before the inhalation—*preferably brandy*, as it is less apt to nauseate than whisky. The surgeons in the Confederate army reported that they had no fatal cases after adopting this practice, but that they did have deaths before they adopted it. They used whisky. Fourth, *never, under* any circumstances, give chloroform to a patient in any other than the recumbent position, lying with the head nearly on a level with the body. Fifth, give the chloroform in weak atmospheres in the beginnig, gradually increasing the strength of the vapor until Anæsthesia is produced, using a towel folded into a hollow cone *with the apex open to admit air.* Sixth, *always stop short of stertorous breathing.* If the breathing is hard, rattling with saliva and mucus in the throat, turn the patients head on its side, at the same time tilting it a little downward toward the shoulder to prevent its flowing down the throat. Great care should always be taken in all cases of Anæsthesia, *produced by whatever agent*, to prevent the blood from running back into the throat, for a few drops getting into the epiglottis might produce death. After Anæsthesia, if there appear to be any indications suggesting a restorative, as feeble pulse, and great paleness of the surface, I crush a small glass globe on a napkin, containing five drops of *nitrate amyl* and let the patient inhale it, which stimulates the heart so that in a few seconds the entire surface assumes the healthy glow of color.

On some occasions I have seen proper to practice Nelaton's method of elevating the extremities and lowering the head when I was apprehensive that the heart's action was too feeble. In observing these simple rules, *I have never had anything resembling an accident.* So great is my confidence in chloroform that I would not care to replace it with any other agent, except for one reason, and that is, it takes so much time for the patient to recover to normal condition, thus monopolizing our attention that is needed by others who are waiting for our services.

For this reason I prefer to use nitrous oxide gas when there are only a few teeth to be removed. But in all cases where it is evident that *it will take time to complete the extractions*, as when there are many fangs and broken teeth, I prefer to place the patient under the *influence of chloroform*—producing gentle but sure sleep, when I turn the head on its side and complete the operations before the Anæsthesia passes off. By this course I control the blood—that is, prevent it from going into the stomach to produce nausea, as as it will invariably do if swallowed. Not long since I read a paper from the pen of Dr. Julian J. Chisholm (whom I consider high authority) on Bromide of Ethyl, in which he gives an experience covering a year or more with this agent for short, painful operations on the eyes, and so strong was his commendation of this agent that I determined to give it a trial at once, in those cases which I ordinarily use gas. I have given it to some fifteen persons and am thus far perfectly charmed with it. It has not the offensive odor of sulphuric ether, produces its effects and passes of as readily as gas, giving ample time for the removal of two or three teeth. Like the *gas*, there must be no air allowed to be breathed—if so, no Anæsthesia will be produced. Take a towel, folding it, with a newspaper between the fold, and make a cone—the paper enclosed in the towel will exclude the air—instruct the patient to take deep drawn inspirations, showing them how to do it beforehand, pour into the cone about one drachm of the Ethyl Bromide and a few inhalations, not exceeding a dozen, will do the work. There is no need to give any stimulant beforehand, as with chloroform. In one of my patients, a lady, there was slight nausea, but I attributed that to the fact, I gave it to her three times in succession (which I ought not to have done) to enable me to remove some fourteeen teeth and fangs. It must be borne in mind *that the inhalation must be pressed upon the patient* in spite of *struggles and resistance, that the admission of air will not prevent Anæsthesia.*

I am indebted to Dr. Chisholm for these instructions, which, if carefully observed, will insure the same splendid results to others.

Some of the members of the Georgia State Dental Society may remember that Dr. J. M. Riggs read a paper before the Society, at its meeting in the city of Atlanta, May, 1880, in which he very strongly urged the use of ethyl bromide. But only a short while before that, Dr. Sims gave a graphic description of the death of the first any only patient to whom he had administered this agent, published in one of the New York Medical Journals: and soon thereafter followed some deaths from its administration in Philadelphia, which placed this agent in the background. Dr. Chisholm has demonstrated, however, that the ethyl bromide was not properly understood, and that it was not suited for long, continued Anæsthesia, life being destroyed, perhaps, by bromide poisoning, while the rapid breathing of a small quantity quickly and safely produces insensibility to pain, and almost as quickly passes off. My experience with this agent, thus far, has given me a preference for it over nitrous oxide gas, for these reasons: First, it is more convenient, requiring no complicated apparatus for its administration. Second, it is inexpensive as compared with the gas. Third, it is equally as safe.

We have demonstrated, to our own satisfaction at least, that we have three (for counting sulphuric ether), Anæsthetic agents capable of overcoming sensibility to pain; either of which, under intelligent administration, is reasonably safe. We have furthermore shown that the way is open for dentists to become *even more skillful* in the administration of these Anæsthetics than the average physician, and the only question to be settled, is it necessary to produce Anæsthetia for extracting teeth! For myself, I answer, yes! If it be true as it has been taught by all writers, and verified by common experience, that *pain is indeed* an evil, that it produces shock and causes reflex action, which is unlimited save by the cessation of life in its effects, in that this may and has been known to result from extracting teeth just as easily as from any other painful operation—if this be true, then the sustaining power of this wonderful Anæsthesia should be given for extracting teeth.

I remember reading in the early fall in some journal, that an old lady in the city of London, had a tooth extracted without any Anæsthetic having been administered, death following immediately after the extraction. The autopsy revealed a diseased heart, and the dentist congratulated himself upon the fact that he had not administered an Anæsthetic, giving as a reason that it would have killed her!

Here was a case, in my opinion, that not only called for, but demanded, Anæsthesia. If she had been placed under the influence of chloroform, in my opinion, she would have passed through the operation safely, and yet living. Why? Because the shock to the nervous system by the extraction would have been entirely prevented by Anæsthesia. Another case reported recently as having occurred in Canada, in which a boy about twelve years old was placed in a dentist's chair and his *family physician administered a small* quantity of chloroform—a tooth was extracted, when the boy struggled so violently that he actually got out of the chair on to the floor, where he remained, breathing hard, and at the expiration of two hours he died.

Now, what does this case teach? It is evident that *Anæsthesia was not produced in this case*—that he was only carried to the first stage—that of excitement, and in his struggles to get away from the dentist, there was undoubtedly a rupture of a small blood-vessel within the cranium

—all the symptoms as described point to this conclusion, and also teaches that *if Anæsthesia had been produced*, there would have been no shock, no struggling and no death. It is my opinion, as well as that of some others who have noted the fatal cases reported, where small quantities of chloroform had been inhaled, that death is from shock, owing to the absence of Anæsthesia. What has been done may be done again, and what is being done by some, may be done by all who qualify themselves to do it; and I speak confidently, as one whose experience entitles him to a profound conviction, that it is cruel and inhuman to deny the comfort of Anæsthesia in extracting teeth. If the majority of dentists are not properly equipped for this duty, as a result of insufficient knowledge of the nature and behavior of Anæsthetic agents, let them set about it at once to gain this knowledge, and attain to the skill in their administration. When they have qualified themselves for the discharge of this high duty, let them have the courage of their convictions, and cease to shirk the responsibility by placing it on the shoulders of the family physician, not one in five hundred of whom are competent to administer Anæsthetics.

I do not mean to reflect upon the physician in the least, I only speak what I believe to be the truth, knowing that after a certain amount of theoretic knowledge of the *modus operandi* of Anæsthetic agents, experience outranks all else, and the average physician rarely attains to this experience. Besides, it seems to me to be an absurb state of affairs, that the dentist should be dependent on an outsider to render assistance necessary for the execution of his legitimate work. Take the following to illustrate this idea: a lady applies to a dentist for the extraction of a tooth, but she will not consent to the operation (it is no argument to her to say it is simple) without Anæsthesia, and the dentist tells her that she must have a physician, and she says she will have none other than her own family physician; it is late in the afternoon, and this particular physician has just gone fifteen miles into the country, thus forcing a delay of twelve or fifteen hours.

Furthermore, it is useless to say that your patients shall not have the benefit of Anæsthesia, *for have it they will*, and if one dentist will not qualify himself to utilize the knowledge that will enable him to " fetter pain," *another will*, and your patients will go from your office to that of one who recognizes his obligations, to boldly meet the giant pain, and with the aid of this blessed knowledge, firmly rivet the " fetters " around his writhing body, thereby cause the despairing cry of agony to be heard in his office no more forever.—*Dental Luminary.*

---

ARTICLE IV.

